# A synergistic bacterial pool decomposes tebuthiuron in soil

**DOI:** 10.1038/s41598-022-13147-8

**Published:** 2022-06-02

**Authors:** Edivaldo Wilson de Lima, Bruno Pinheiro Brunaldi, Yanca Araujo Frias, Bruno Rafael de Almeida Moreira, Lucas da Silva Alves, Paulo Renato Matos Lopes

**Affiliations:** Department of Plant Production, College of Agricultural and Technological Sciences, São Paulo State University (UNESP), Dracena, SP 17900-000 Brazil

**Keywords:** Biological techniques, Microbiology

## Abstract

This study aimed to propose an eco-compatible strategy to mitigate the possible environmental contamination caused by tebuthiuron. Therefore, we screened potential tebuthiuron-degrading microorganisms from conventional (CS) and no-till (NTS) systems producing sugarcane. Then, they were bioprospected for their ability of decomposing the target-molecule at 2.48 mmol g^−1^ and 4.96 mmol g^−1^ into CO_2_ via respirometry. Integrating microbiota from CS and NTS into an advantageously synergistic bacterial pool produced the highest specific-growth rate of CO_2_ of 89.60 mg day^−1^, so outstripped the other inoculum. The bacterial CN-NTS framework notably stabilized the sigmoidal Gompertz curve on microbial degradation earliest and enabled the seeds of *Lactuca* *sativa* to germinate healthiest throughout ecotoxicological bioassay for cross-validation. Our study is preliminary, but timely to provide knowledge of particular relevance to progress in the field's prominence in remediating terrestrial ecosystems where residual tebuthiuron can persist and contaminate. The analytical insights will act as an opening of solutions to develop high-throughput biotechnological strategies for environmental decontamination.

## Introduction

**Figure 1:**
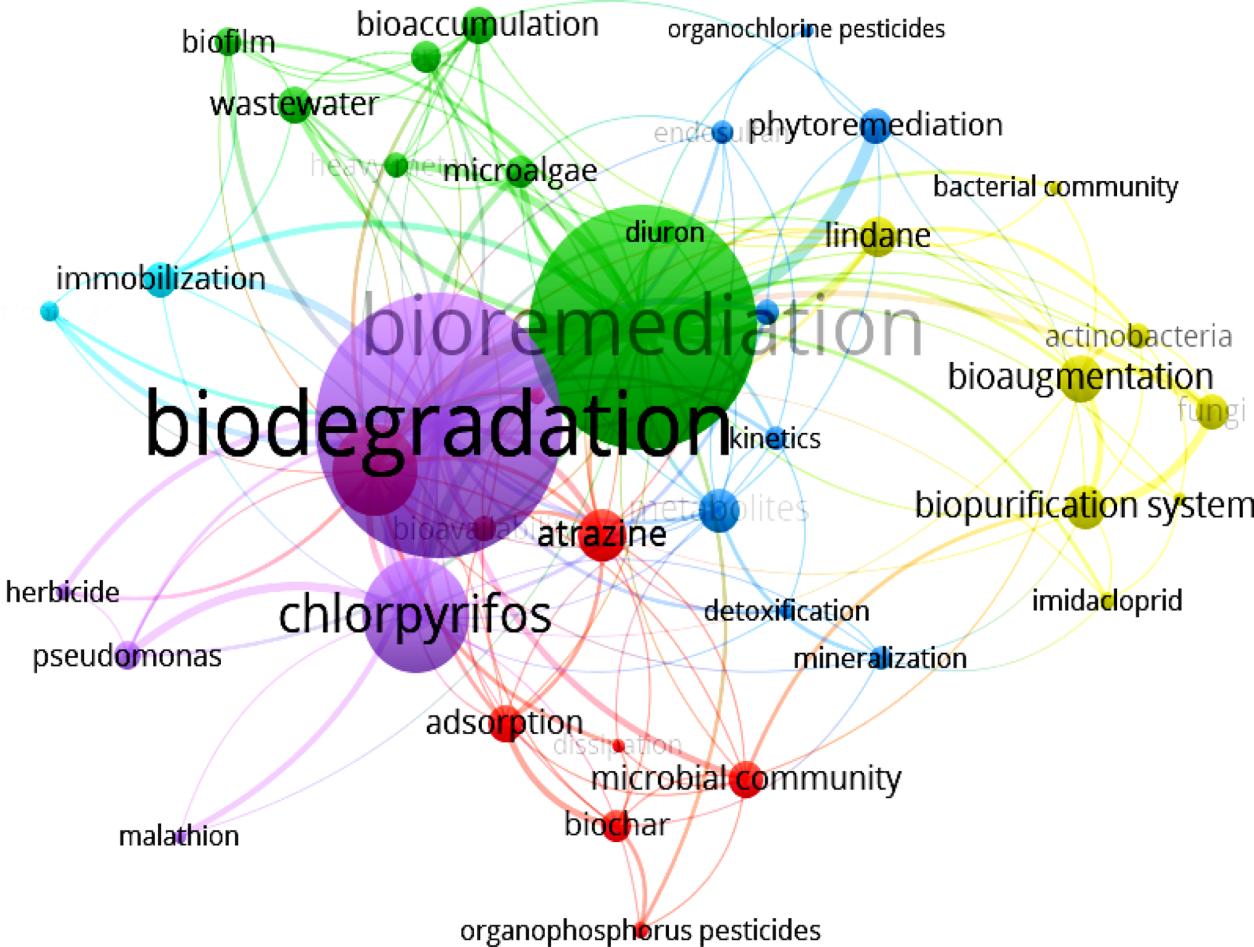
Co-occurrence of keywords on the specific topic of “pesticide bioremediation” (*Scopus,* *Web* *of* *Science*, and *Wiley* *Online* *Library* on 12th April, 2021).

By searching for the academic specific topic of "pesticide bioremediation", we can screen-out numerous harmful molecules. Chlorpyrifos, malathion, atrazine, lindane and imidacloprid have greater intellectual interest by researchers and science policymakers than any other active compound (Fig. 1). All of them are sources of neurotoxins to pollinizers and invertebrates^1,2^. However, no single in-depth study exists for the microbiological detoxification or mineralization of tebuthiuron in soil.

Tebuthiuron is the dominant member of phenylurea^3^. It is useful to control grassy and broadleaf weeds in areas producing sugarcane. However, it is highly water-soluble (2.50 g L^−1^) and can escape easily into ecosystems, where non-target organisms do not resist xenobiotics^4,5^. Carryover of biotoxins by residual tebuthiuron leaching can promote contamination and loss of biodiversity by food chain bioaccumulation or environmental exposure^6,7^.

Although tebuthiuron is not the focus of literature on pesticide depollution, we can find few contemporary examples of its successful bioremediation. Mendes et al.^8^ when studying the phytoremediation of pesticides by green manure, reported the ability for *Crotalaria* *spectabilis*, *Canavalia* *ensiformis*, *Stizolobium* *aterrimum*, and *Lupinus* *albus* to effectively remove C-tebuthiuron at 266.40 g ha^−1^. *Mucuna* *pruriens* and *Pennisetum* *glaucum* were also able to dissipate C-tebuthiuron at 500 g ha^−1^ in soil with stillage as an organic matter to boost performance^9^. The authors^9^ cross-validated the potential phytoremediators by checking the normal germination of an organism sensible to the target-molecule throughout ecotoxicological bioassay.

Thus, phytoremediation proves useful to remediate tebuthiuron. However, it often requires a special management and makes it challenging for agricultural systems to produce food, energy and natural fiber in off-season^8,9^. Furthermore, it is not easy to simulate conditions on an industrial scale. Therefore, an option to compensate the complexities of phytoremediation would be microbial degradation.

Microbial degradation is the major route of dissipation for photosystem II herbicides in aquatic ecosystems^2^. Bacteria can effectively degrade diuron, atrazine, hexazinone and tebuthiuron in seawater, with straightforward evidence on hydrolysis as the predominant pathway^10^. However, the authors^2,10^ highlighted the importance of reproducing systematic studies for clarity and, most notably, analyzing environments other than coastal waters to progress in the field’s prominence in microbiologically dissipating pesticides.

To the best of our knowledge, no in-depth investigation exists on the potential of microorganisms to remediate tebuthiuron in soil. Therefore, in light of research and innovation in pesticide-remediating eco-solutions, the novelty of our paper refers to the elaboration of a synergistic bacterial pool to dissipate tebuthiuron out of agricultural soil. Our exploratory study is still at an early stage of development. However, preliminary analytical insights into respirometric-ecotoxicological ramifications of microbial biotransformation of the target-pollutant into a simpler and harmless compound are timely. Our remedial approach is effective and will be likely useful for the purpose of environmental recovery in agroecosystems, such as intensive sugarcane producing areas, where tebuthiuron acts as a highly toxic and recalcitrant pollutant and disrupts the agronomic functionalities besides the ecological sustenance.

## Material and methods

### Target-pesticide and microbial isolates

**Figure 2:**
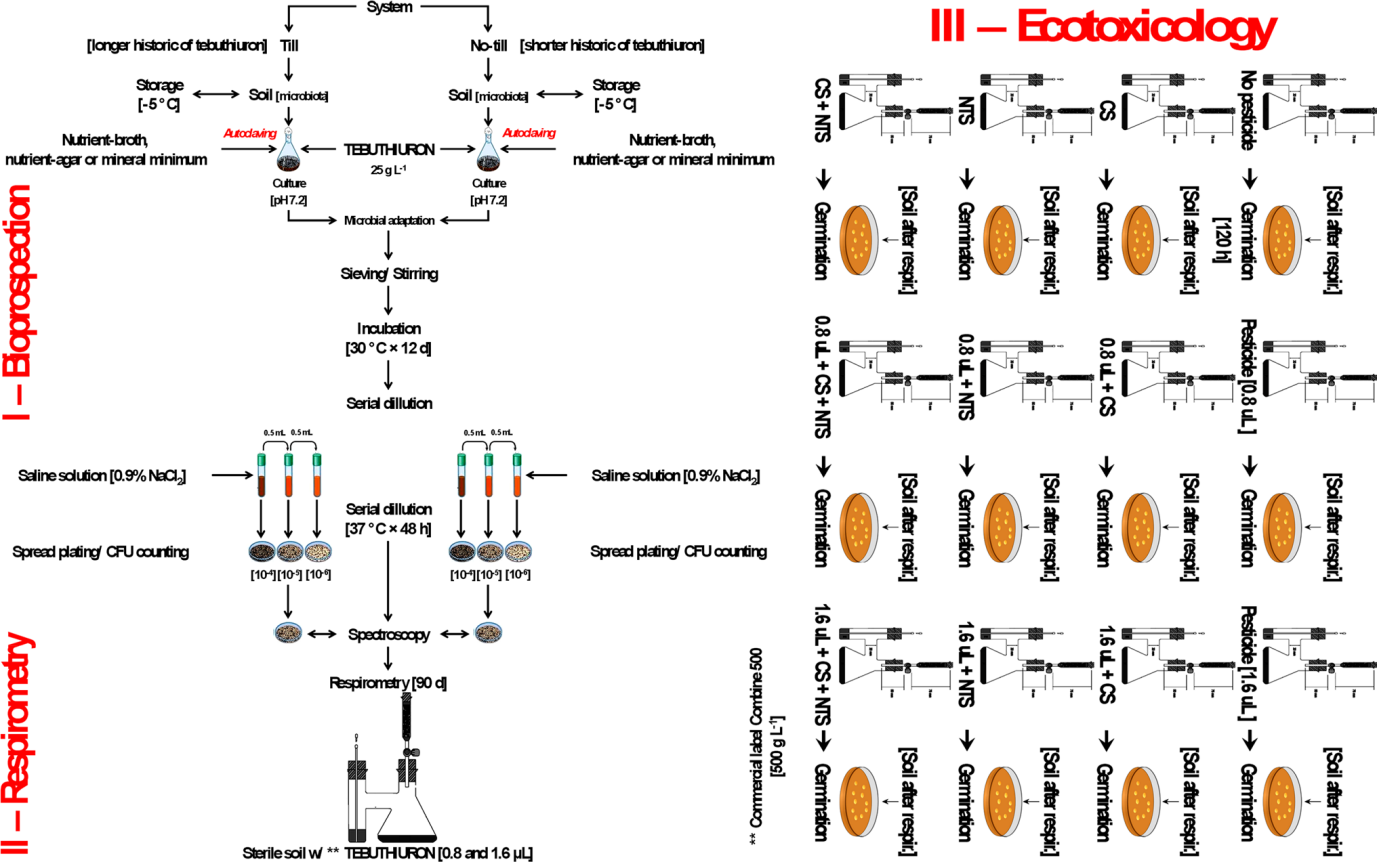
Scheme of isolating native microorganisms to mineralize tebuthiuron into CO_2_. In the diagram, the doses of 0.8 µL and 1.6 µL are equivalent to the concentrations of 2.48 mmol g^−1^ and 4.96 mmol g^−1^ in the soil (half-dose and full-dose of tebuthiuron, respectively.

The pesticide used was tebuthiuron (Combine 500, Down AgroSciences Industrial). We isolated the potential tebuthiuron-degrading native microorganisms from conventional (longer historic of tebuthiuron) and no-till (shorter historic of tebuthiuron) systems producing sugarcane. Both sites were treated with the broad-spectrum herbicide at 800.00 g kg^−1^. The in-situ microbial bioprospection consisted of randomly sampling five points at 0.00–0.15 m depth around the radicles. Then, the soil was transferred to air-tight bags. The material was stored in freezer at −5.00 °C to cryopreservation until further laboratorial procedures of spread plating and inoculation (Fig. 2).

### Inoculum preparation

The nutrient-broth, nutrient-agar and mineral minimum broth were autoclaved (121 °C × 0.25 h) to formulate selective culture media. Particularly the mineral minimum broth consisted of 0.70 g KCl, 0.20 g KH_2_PO_4_, 3.00 g Na_2_HPO_4_, and 1.00 g NH_4_NO_3_ per liter, with an additional 1.00 mL L^−1^ solution of micronutrients: 4.00 mg MgSO_4_, 0.20 mg FeSO_4_, 0.20 mg MnCl_2_, and 0.20 mg CaCl_2_^11^. Mimetic microcosms were elaborated by introducing 25.00 g L^−1^ of tebuthiuron into all media at pH 7.20 in order to adapt the isolates to the pesticide until pre-selection. Aliquots of 90.00 mL were then thoroughly mixed with 50.00 g of soil (2 mm granulometry) in Erlenmeyer flasks and were mechanically stirred at 120.00 rpm, 30.00 °C for 72.00 h for homogenization. The material was incubated at room temperature for 12 days. After incubation, 0.50 mL of all microbial suspensions was diluted in sterile saline solutions (0.90% NaCl) to prevent cross-contamination. Aliquots of 1.00 mL were evenly streaked out over nutrient-agar plates at 10^–4^, 10^–5^ and 10^–6^ and stored at 37 °C for 48.00 h. Finally, we automatically counted the colony-forming units on the surface of plates to check cellular viability. Thereafter, we performed spectrophotometric measurements at 600.00 ηm^12^ to standardize inoculum at 0.80 absorbance unit for the respirometric bioassay.

### Respirometric bioassay

A respirometric bioassay was performed to quantitatively analyze the ability of the isolates to mineralize tebuthiuron into CO_2_, according to methodology described by Bartha and Pramer^13^. Samples of soil (Table S1, Supplementary material) were collected at the layer of 0.00–0.30 m in the experimental field of the Plant Production Division of the College of Agricultural and Technological Sciences, São Paulo State University (Unesp). The material was oven dried at 65 °C until constant mass, sieved to 2.00 mm granulometry and autoclaved at 121.00 °C for 0.25 h for sterilization before preparing media to the respirometry. Aliquots of 0.05 kg of sterile soil and tebuthiuron at 0.8–1.6 µL were introduced together into respirometers and then the isolates were inoculated. After inoculation, 10.00 mL of KOH at 1 M were transferred to flasks to capture the production of CO_2_ upon the target-pesticide at the concentrations of 2.48 mmol g^−1^ and 4.96 mmol g^−1^ as the half-dose and full-dose, respectively. We quantified the product of microbial respiration by electroconductivity of CO_3_^–2^ (Eq. 1) every day after titrating the KOH with 10.00 mL of BaCl_2_ at 1 M. After quantification, 10.00 mL of KOH was added to respirometers for the next samples^13^. The bioassay lasted for 90 days. Flasks were incubated at 25.00 ± 2.50 °C and 60.00 ± 5.00% of relative humidity of the air, in dark room to prevent photodecomposition^10^. The set was aerated for renewing atmosphere every day after quantifying the production of CO_2_ from the experimental units (Table 1) in triplicate to reduce systematic errors.

1 $$G=1554.80-95.7O C$$

where: G is the production of CO_2_, mg; C is the electroconductivity, mS cm^−1^; and the constants stand as factors of transformation of concentration of the analyte from mmol to mg^13^.

**Table 1:** Set of tests for the respirometric bioassay of potential microbial biodegradation of tebuthiuron.

Test	Tebuthiuron, mmol g^−1^	Source of native microbiota	
		Conventional system	No-till system
I	No pesticide	−	−
II	2.48, half-dose	−	−
III	4.96, full dose	−	−
IV	No pesticide	+	−
V	2.48, half-dose	+	−
VI	4.96, full dose	+	−
VII	No pesticide	−	+
VIII	2.48, half-dose	−	+
IX	4.96, full dose	−	+
X	No pesticide	+	+
XI	2.48, half-dose	+	+
XII	4.96, full dose	+	+

### Ecotoxicological bioassay

To cross-validate our approach, ecotoxicity of tebuthiuron after an eventual microbial bioremediation was determined in triplicate in seeds of a utilitarian organism (*Lactuca* *sativa*) based on experimental protocols. Samples of soil (0.0025 kg) were collected from flasks at the beginning (t_0_) and the end (t_90_) of the respirometric bioassay. Twenty-five seeds were randomly selected, then evenly distributed over acrylic plates to have contact with the xenobiotic. The plates were incubated in biochemical oxygen chamber at 25.00 ± 2.50 °C, 50.00 ± 5.00% relative humidity, and 16:8 h of photoperiod. The ecotoxicological bioassay lasted for 120.00 h, and the tests were sampled every 24.00 h to quantify the germination and radicle-to-hypocotyl ratio as indicators of vigor and phenotypical morphophysiology, respectively^14^. Media with water and zinc sulphate as an inhibitor of germination were prepared as positive and negative controls, respectively.

### Data analysis

We fitted the data on respirometry and ecotoxicology for sigmoidal Gompertz function (Eq. 2), starting the parametrization with α = 1000, β = 10 and κ = 0.50^15^. The criteria to analyze the adequacy of the stochastic model included Akaike information criterion (AIC), Bayesian information criterion (BIC), and adjusted coefficient of determination (r_adj_^2^).

2 $${f}_{x}= \alpha {e}^{-\beta {e}^{-kx}}$$

where: *f*_*x*_ is the production of CO_2_ or germination, mg or %; *x* is the time, days; *α* is the upper asymptote or the maximum of production of CO_2_ or germination, mg or %; *β* is the inflection point; *κ* is the exponential decay of specific-growth rate of production of CO_2_ and germination, mg CO_2_ day^−1^ and % day^−1^; and e is the Euler number.

A box-plot diagram was elaborated to describe the radicle-to-hypocotyl ratio for samples from ecotoxicological bioassay, separating them using the *post-hoc* Tukey’s test. Principal component analysis^16^ was conducted to extract functional relationships between respirometry and ecotoxicology. All analyses were performed in the environment of the software R-project for statistical computing and graphics^17^.

### Ethics approval

Authors confirm that the manuscript has not been submitted to journal for simultaneous consideration and has not been previously published. Results collection, selection, and processing performed personally. Authors’ institution informed about this submission.

### Consent for publication

All authors approved the manuscript before submission and consent to the submission to *Scientific* *Reports*.

## Results

### Morphological characterization of isolates

The isolates, irrespective of origination, consisted of bacterial colonies. Mineral broth medium produced more colony-forming units than nutrient-broth and nutrient-agar. Therefore, it proved the most reliable option to culture potential tebuthiuron-degrading bacteria.

### Kinetic mineralization

**Figure 3:**
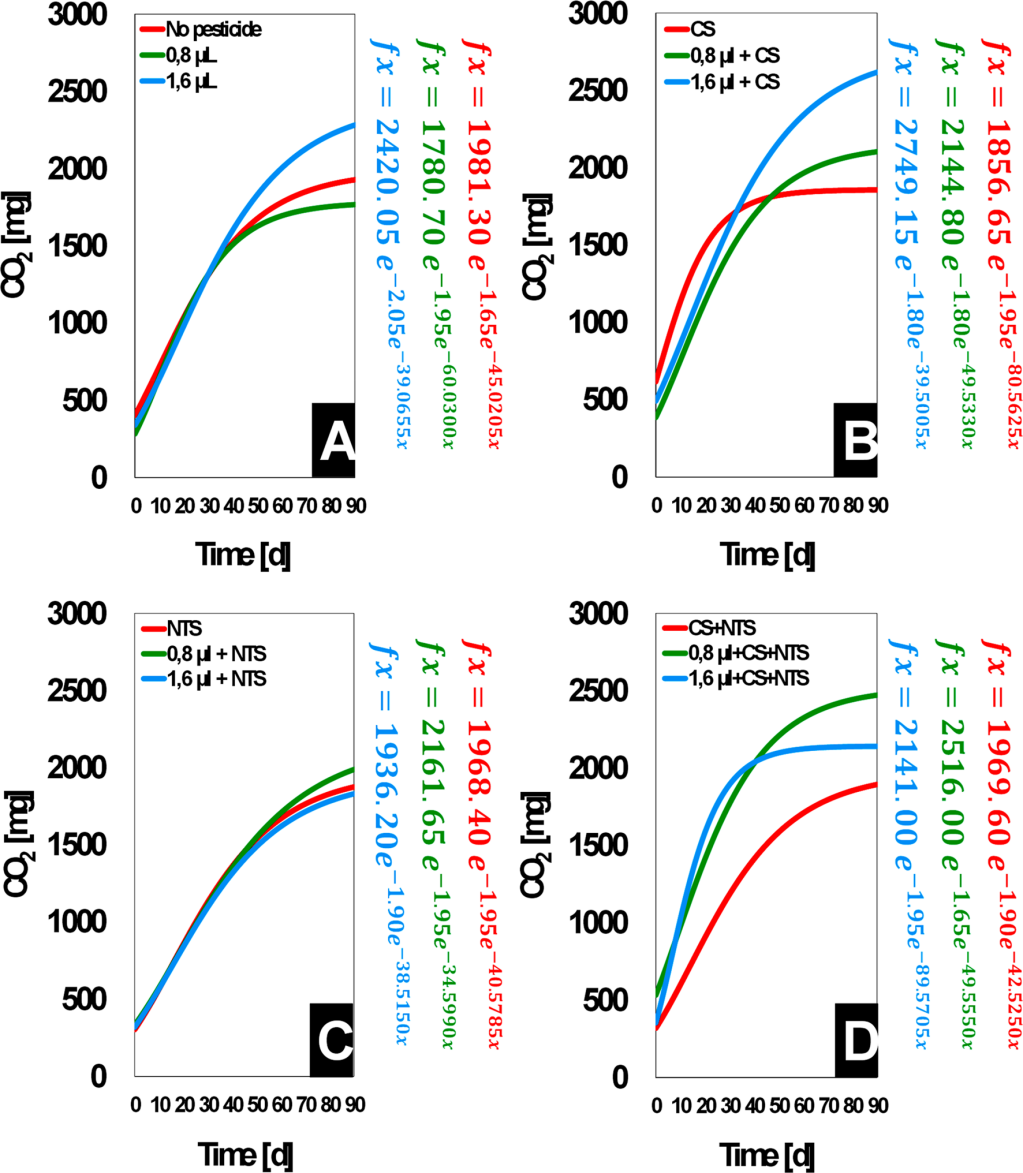
Sigmoidal mineralization of tebuthiuron in media containing only soil (**A**) and soil with isolates from conventional system (**B**) and no-till system (**C**) and both (**D**). In the diagram, the doses of 0.8 µL and 1.6 µL are equivalent to the concentrations of 2.48 mmol g^−1^ and 4.96 mmol g^−1^ in the soil (half-dose and full-dose of tebuthiuron, respectively); Conventional (CS) and no-till (NTS) systems; *β* = 1 keeps the relative decrease with time constant; *β* > 1 accelerates the relative decrease with time; *β* < 1 decelerates the relative decrease with time^15^.

Integrating CS and NTS into a bacterial pool enhanced the sigmoidal mineralization of tebuthiuron into CO_2_ (Fig. 3D). Plainly, composite inoculum proved synergistic effect and, hence, outstripped both CS and NTS in stabilizing the Gompertz breakthrough curve for the target-pesticide at 4.96 mmol g^−1^ (Fig. 3D). The bacterial CS-NTS framework required about 24 days to approach the maximum value for biotransformation of tebuthiuron at the highest dose into CO_2_ (*α* ~ 2141.00 mg) (Table 2), and our stochastic analysis on Gompertz adequately predicted its ability for accelerating the process into an inflection point of 1.95 and relative growth-rate of 89.60 mg CO_2_ day^−1^. Comparatively, values of *β* and *k* for CS and NTS were 1.80 and 39.50 mg CO_2_ day^−1^ (Fig. 3B) and 1.90 and 38.50 mg CO_2_ day^−1^ (Fig. 3C), respectively, for 4.96 mmol g^−1^. For the lowest dose, we could parametrize 1.80 and 49.55 mg CO_2_ day^−1^ and 1.95 and 34.60 mg CO_2_ day^−1^, respectively. Therefore, both CS and NTS required longer periods of time to reach the maximum relative value to CS-NTS. As CS (*β* = 1.95; k = 80.5625 mg day^−1^) (Fig. 3B) generated CO_2_ more intensively than NTS (*β* = 1.95; k = 40.5785 mg day^−1^) (Fig. 3C), it likely contributed more to the ability of the composite inoculum to accelerate the sigmoidal mineralization throughout 90 days respirometry. If CS-NTS proved synergistic effect towards skewing upwards the breakthrough curve for the mineralization of tebuthiuron at 2.48 mmol g^−1^ (Fig. 3D), a flatter and longer stationary phase on Gompertz function for its performance at 4.96 mmol g^−1^ followed the dependence of growing bacterial population on concentration. Most notably, curves for CS-NTS at 2.48–4.96 mmol g^−1^ steeper than its contrasting curve without the pesticide (Fig. 3D) supported the role of tebuthiuron in providing the bacterial growth by acting as a source of mineralizable carbon, besides the adequacy of Gompertz to describe growth sigmoidal rather than linear. Furthermore, tebuthiuron was not necessarily the only source of energy to microorganisms, as it released CO_2_ (Fig. 3A) without introducing isolates, irrespective of origination, into the soil for respirometry.

**Table 2:** Parametrization and adequacy of Gompertz model for the kinematic microbial biodegradation of tebuthiuron.

Test	Parameterization			Adequacy		
	*α*, mg	*β*, days	*κ*, mg day^−1^	AIC	BIC	r_adj_^2^
No pesticide	1981.30	1.65	45.0205	364.2270	369.8315	0.9540*
2.48 mmol g^−1^	1780.70	1.95	60.0300	335.6625	341.2670	0.9465*
4.96 mmol g^−1^	2420.05	2.05	39.0655	360.8330	366.4380	0.9450*
CS	1856.65	1.95	80.5625	334.5820	340.1865	0.9810*
2.48 mmol g^−1^ + CS	2144.80	1.80	49.5330	323.4520	329.0570	0.9905**
4.96 mmol g^−1^ + CS	2749.15	1.80	39.5005	354.0185	359.6230	0.9825*
NTS	1968.40	1.95	40.5785	290.5405	296.5410	0.9960**
2.48 mmol g^−1^ + NTS	2161.65	1.95	34.5990	317.3815	322.9865	0.9910**
4.96 mmol g^−1^ + NTS	1936.20	1.90	38.5150	298.4065	304.0115	0.9945**
CS + NTS	1969.60	1.90	42.5250	313.4130	319.0175	0.9920**
2.48 mmol g^−1^ + CS + NTS	2516.00	1.65	49.5550	369.1560	374.7610	0.9655*
4.96 mmol g^−1^ + CS + NTS	2141.00	1.95	89.5705	365.3595	370.9640	0.9725*

2.48 and 4.96 mmol g^-1^ are half-dose and full-dose of tebuthiuron, respectively.

*CS* conventional system, *NTS* no-till system, *AIC* Akaike information criterion, *BIC* Bayesian information criterion.

Significant code: **p < 0.01; *p < 0.05.

### Ecotoxicology

**Figure 4:**
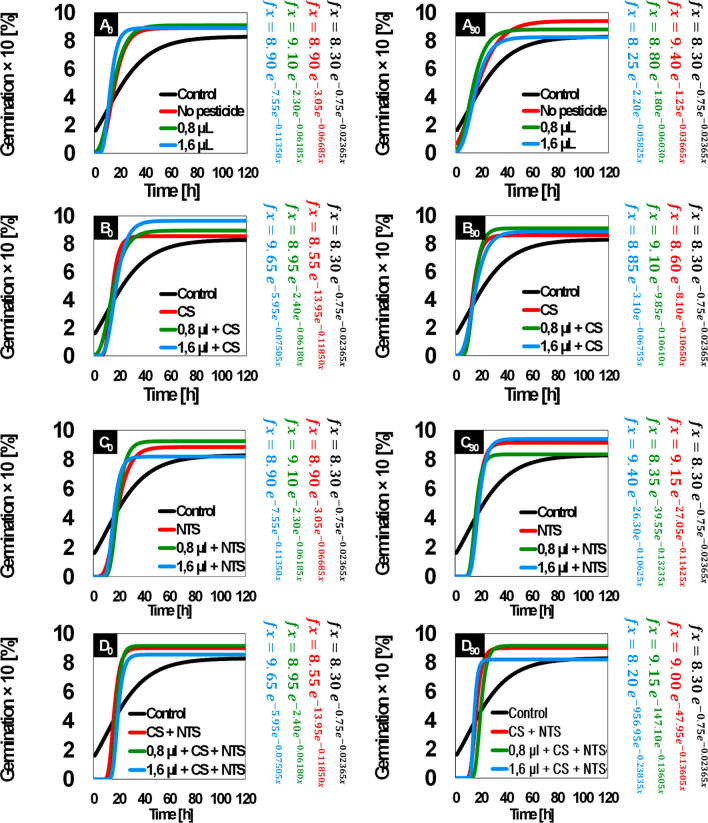
Sigmoidal germination of an organism sensitive to tebuthiuron on plates containing respirometric samples of only soil (**A**) and soil with isolates from conventional system (**B**) and no-till system (**C**) and both (**D**). In the diagram, the doses of 0.8 µL and 1.6 µL are equivalent to the concentrations of 2.48 mmol g^−1^ and 4.96 mmol g^−1^ in the soil (half-dose and full-dose of tebuthiuron, respectively); Conventional (CS) and no-till (NTS) systems; Left-panel (*t*_*0*_*—*initial time) and right-panel (*t*_*50*_*—*after 90 days of biodegradation); *β* = 1 keeps the relative decrease with time constant; *β* > 1 accelerates the relative decrease with time; *β* < 1 decelerates the relative decrease with time^15^.

**Figure 5:**
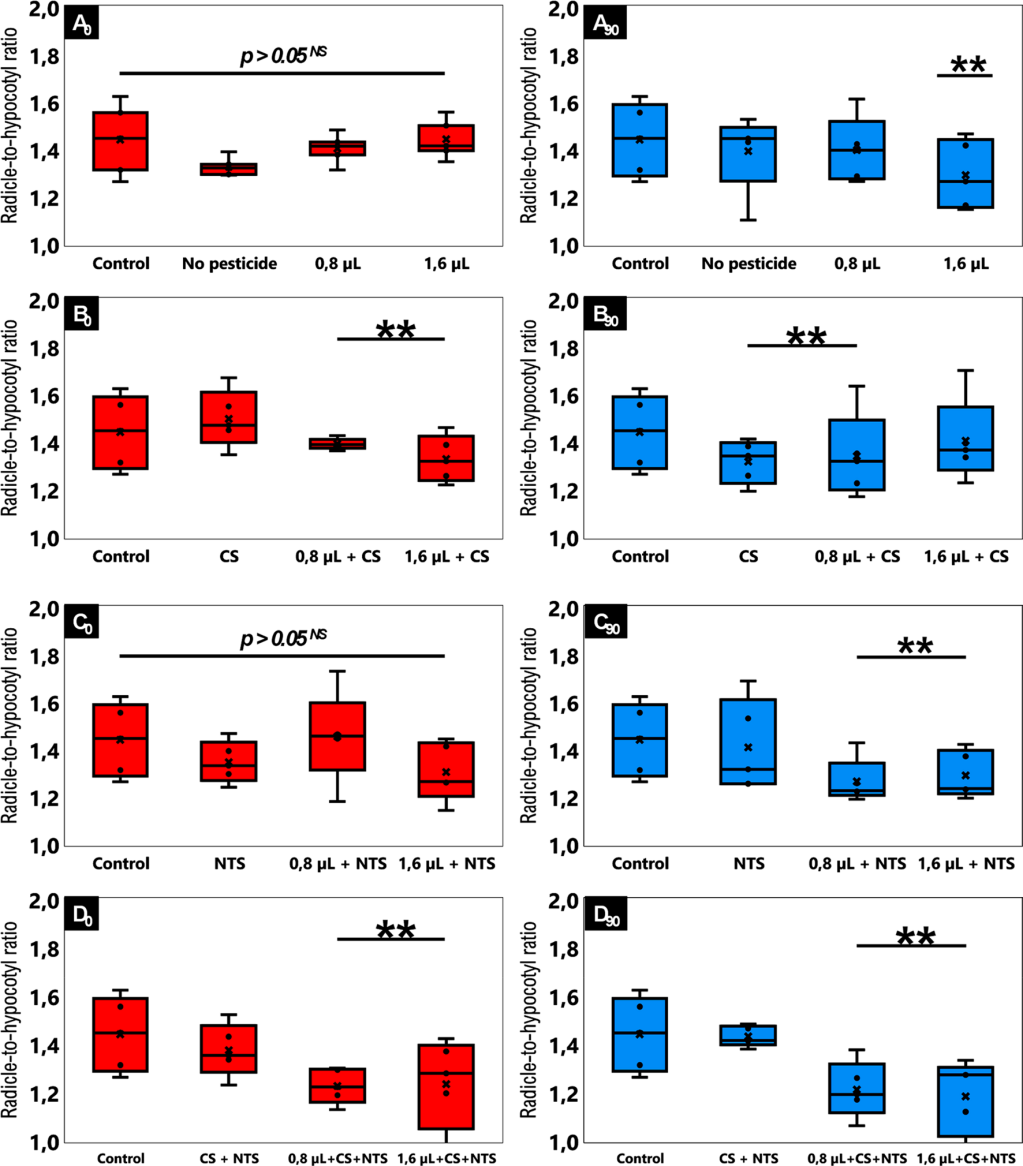
Radicle-to-hypocotyl ratio of an organism sensitive to tebuthiuron on plates containing respirometric samples of only soil (**A**) and soil with isolates from conventional system (**B**) and no-till system (**C**) and both (**D**). In the diagram, the doses of 0.8 µL and 1.6 µL are equivalent to the concentrations of 2.48 mmol g^−1^ and 4.96 mmol g^−1^ in the soil (half-dose and full-dose of tebuthiuron, respectively); *Significant and *NS* non-significant by post hoc Tukey’s test at p < 0.05; conventional (CS) and no-till (NTS) systems; left-panel (*t*_*0*_*—*initial time) and right-panel (*t*_*50*_*—*after 90 days of biodegradation).

Since bacterial CS-NTS framework most effectively mineralized the herbicide at 2.48 mmol g^−1^ into CO_2_, it enabled the seeds to have the highest percentage germination over time (Fig. 4, D0 and D90). In addition, seedlings developed fastest and healthiest with radicle-to-hypocotyl ratio closest to 1 (Fig. 5, D0 and D90). The best result of the CS-NTS microbial consortium can be observed when comparing with the results of the *Lactuca* *sativa*’s germination index in relation to the presence of isolated inoculums CS (Fig. 4, B0 and B90) and NTS (Fig. 4, C0 and C90). Consequently, the same relationship can be performed with the radicle-to-hypocotyl ratio of this organism sensitive to tebuthiuron for isolates CS (Fig. 5, B0 and B90) and NTS (Fig. 5, C0 and C90).

However, ecotoxicological units containing soil from respirometers without microbiota, irrespective of composition, delayed the germination (Fig. 4, A0 and A90) and induced an atypical radicle-to-hypocotyl ratio of ≥ 1.45 (Fig. 5, A0 and A90). Sensitive organism also proved to be able to germinate on plates containing only tebuthiuron (Fig. 4, A0 and A90). Certainly, the addition of 2.48 mmol g^−1^ and 4.96 mmol g^−1^ tebuthiuron in soil were not sufficient to prevent seed germination. However, these concentrations induced longer and hairier radicle and shorter hypocotyl for *L.* *sativa* (Fig. 5, A0 and A90). These results indicated developmental abnormalities or adaptations to the challenging microenvironment for the seedlings. By fitting the sigmoidal Gompertz function to the germination data, we could estimate *β* > 1 for all tests except negative control containing water (Table 3). A *β* ≥ 1 is an indicator that the growth relative decreasing accelerates with time. On the other hand, *β* < 1 decelerate the growth relative decrease, while the *β* = 1 keeps it constant. Inflection point was greatest for bacterial CS-NTS framework (*β* = 956.95), so seeds germinated and developed fastest into seedlings on plates containing soil from respirometric flasks where concentration of tebuthiuron initially was 4.96 mmol g^−1^ (Fig. 4, D90). Relative growth-rate also was the largest for the CS-NTS (*k* = 0.23835), further supporting its distinct ability for detoxifying the medium. Overall, applying Gompertz to ecotoxicological bioassay, it was possible to validate the effectiveness of the synergistic bacterial pool to mineralize the target-molecule into CO_2_ throughout 90 days respirometry, which potentially make the substrate less harmful. Hence, we could verify that the organism germinated and developed throughout 5 days without any critical abnormality, despite its sensitivity to tebuthiuron.

**Table 3:** Parametrization and adequacy of Gompertz model for kinetic germination of an organism sensible to tebuthiuron on plate containing soil from respirometric bioassay.

Test	Parameterization			Adequacy		
	*α* × 10, %	*β*	*κ* × 10, % h^−1^	AIC	BIC	r_adj_^2^
**Initial time (t**_**0**_**)**						
Control	8.30	0.75	0.02365	−5.90	−7.50	0.9955 **
No pesticide	8.90	3.05	0.06685	3.85	2.30	0.9895 *
2.48 mmol g^−1^	9.10	2.30	0.06185	−2.55	−4.10	0.9960 **
4.96 mmol g^−1^	8.90	7.55	0.11350	−4.45	−6.00	0.9970 **
CS	8.55	13.95	0.11850	5.40	3.80	0.9900 **
2.48 mmol g^−1^ + CS	8.95	2.40	0.06180	−3.70	−5.25	0.9970 **
4.96 mmol g^−1^ + CS	9.65	5.95	0.07505	0.65	-0.92	0.9975 **
NTS	8.85	5.10	0.06500	2.90	1.30	0.9955 **
2.48 mmol g^−1^ + NTS	9.25	20.55	0.09630	−3.70	−5.25	0.9995 **
4.96 mmol g^−1^ + NTS	8.20	24.45	0.11885	0.55	−1.00	0.9975 **
CS + NTS	9.00	47.95	0.13535	0.90	−0.65	0.9985 **
2.48 mmol g^−1^ + CS + NTS	9.15	352.35	0.16850	−4.80	−6.35	0.9995 **
4.96 mmol g^−1^ + CS + NTS	8.55	188.40	0.14570	−6.20	−7.75	0.9995 **
**Final time (t**_**90**_**)**						
No pesticide	9.40	1.25	0.03665	1.40	−0.15	0.9915 **
2.48 mmol g^−1^	8.80	1.80	0.06030	−19.40	−20.95	0.9990 *
4.96 mmol g^−1^	8.25	2.20	0.05825	−17.00	−18.55	0.9995 **
CS	8.60	8.10	0.10650	−22.55	−24.10	0.9995 **
2.48 mmol g^−1^ + CS	9.10	9.85	0.10610	1.95	0.40	0.9950 **
4.96 mmol g^−1^ + CS	8.85	3.10	0.06755	−3.85	−5.40	0.9975 **
NTS	9.15	27.05	0.11425	−7.75	−9.30	0.9995 **
2.48 mmol g^−1^ + NTS	8.35	39.55	0.13235	−5.20	−6.75	0.9995 **
4.96 mmol g^−1^ + NTS	9.40	26.30	0.10625	−11.95	−13.55	0.9995 **
CS + NTS	9.00	47.95	0.13535	0.90	−0.65	0.9985 **
2.48 mmol g^−1^ + CS + NTS	9.15	147.10	0.13605	−8.00	−9.60	0.9995 **
4.96 mmol g^−1^ + CS + NTS	8.20	956.95	0.23835	−46.80	−48.35	0.9995 **

2.48 and 4.96 mmol g^−1^ are half-dose and full-dose of tebuthiuron, respectively.

*CS* conventional system, *NTS* no-till system, *AIC* Akaike information criterion, *BIC* Bayesian information criterion.

Significant code: **p < 0.01; *p < 0.05.

### Mineralization-ecotoxicology nexus

**Figure 6:**
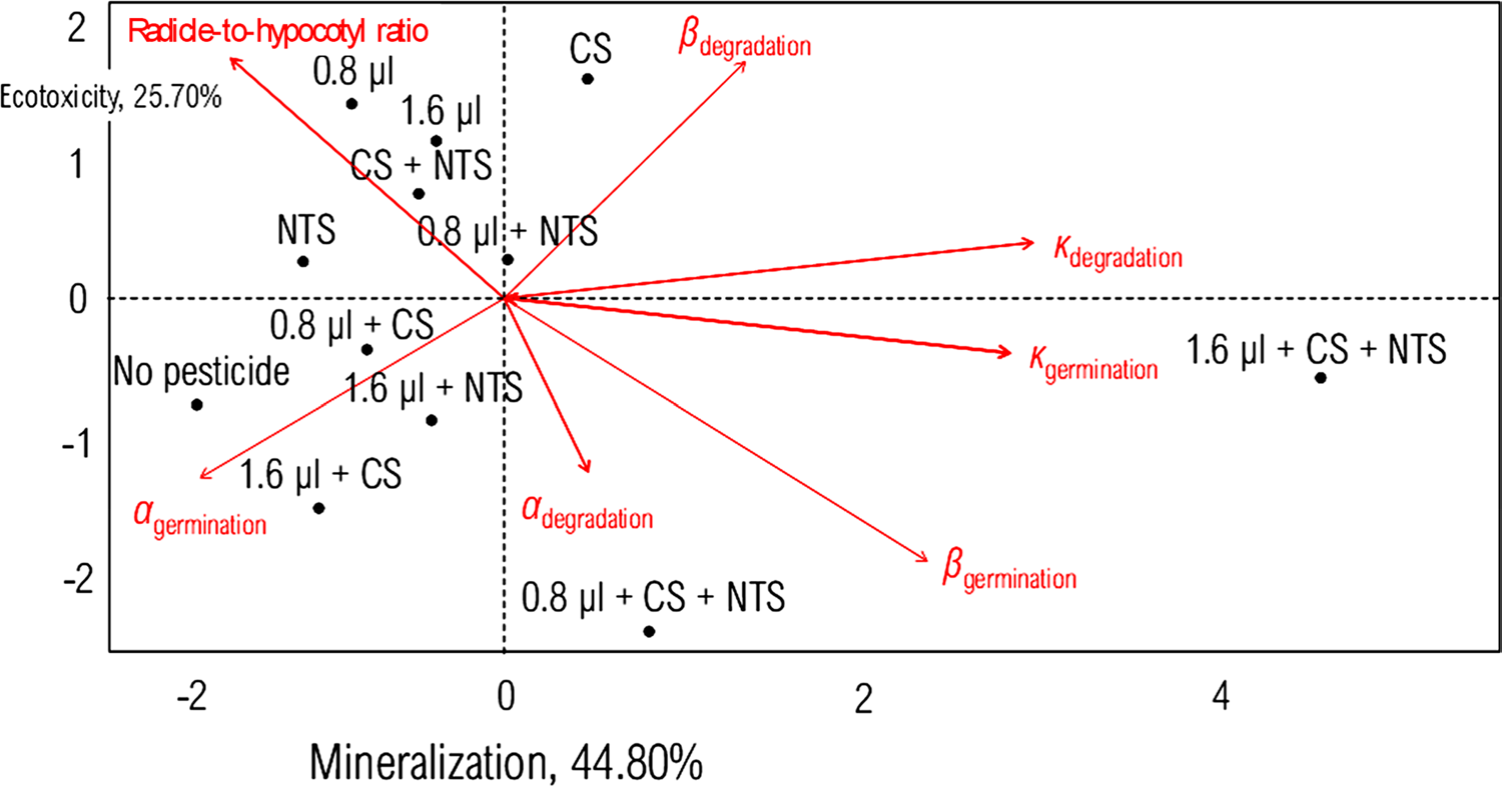
Bi-plot map for the mineralization-ecotoxicology nexus. In the diagram, the doses of 0.8 µL and 1.6 µL are equivalent to the concentrations of 2.48 mmol g^−1^ and 4.96 mmol g^−1^ in the soil (half-dose and full-dose of tebuthiuron, respectively); (CS) conventional and no-till (NTS) systems.

The PCA robustly divided the high-dimensionality dataset and exported only the useful statistics into the latent orthogonal hits, namely PC_I_ and PC_II_ (Fig. 6). The two PC together explained about 70% of variability in the interdependent respirometric and ecotoxicological bioassays (Table 4). The PC_I_ explained the mineralization. It correlated positively with the specific-growth rate of both biodegradation and germination. However, it correlated negatively with the radicle-to-hypocotyl ratio. On the other hand, the PC_II_ explained the ecotoxicity. It correlated positively with both inflection of biodegradation and radicle-to-hypocotyl ratio. The bacterial CS-NTS framework moved towards the left lower quadrant in the bi-plot map, further supporting its effectiveness to mineralize tebuthiuron and make it less toxic over seeds. Therefore, the multivariate analysis of data validated the effectiveness of the synergistic bacterial pool to remediate the target-molecule.

**Table 4:** Principal components into respirometric and ecotoxicological bioassays.

Eigenvector	Latent orthogonal hit	
	PC_I_, mineralization	PC_II_, ecotoxicology
Eigenvalue	2.85*	1.65*
Percentage of variance	44.75	25.70
Cumulative percentage of variance	44.75	70.45
	**Correlation**	
Maximum degradation	0.05	−0.55
Inflection of degradation	0.25	0.85*
Specific-growth rate of degradation	0.75*	0.20
Maximum germination	−0.60*	−0.50
Inflection of germination	0.95**	−0.15
Specific-growth rate of germination	0.90 **	−0.10
Root-to-hypocotyl ratio	-0.65 *	0.65*

*CS* conventional system, *NTS* no-till system.

Significant code: **p < 0.01; *p < 0.05.

## Discussion

Native microorganisms from sugarcane's rhizosphere can be useful to bioremediate the tebuthiuron in soil. By integrating microbiota from conventional and no-till systems into an advantageously synergistic bacterial pool, we can optimize the biotransformation of the target-pesticide into CO_2_. The higher the dose the more effective the mineralization, as the xenobiotic likely becomes more bioavailable from the substrate. Plainly, bioavailability is primordial to bioremediation. If contaminant is available, microorganisms are able to mineralize the carbon^18^. If not, they cannot access the target-molecule, so an effective bioremediation is not likely. The bacterial CS-NTS framework can mineralize the tebuthiuron at half-dose more effectively than CS and NTS, making it an option to compensate the insufficient either electronic donation/acception or systematic stimulation, which are limiting factors to pesticide-metabolizing enzymes^19^.

Isolates from conventional system can adapt to the mimetic microcosm more effectively than isolates from no-till system, supporting our hypothesis of the impact of environmental pressure on the microbial behavior. A longer historic and more frequent applications of tebuthiuron can upregulate the ability for autochthone microorganisms to resist mimetic microcosm containing the pesticide as an elemental component. Therefore, they can accelerate the lag phase for the composite inoculum. The shorter the lag phase the more efficient the microbiota in mineralizing tebuthiuron in the log or exponential phase. Production of CO_2_ is the largest in the log phase and gradually decreases as the biotransformation levels off. Thus, we could find no significant activity for the stationary phase.

Studies on microbial degradation of tebuthiuron focus on aquatic ecosystems^2,10^, making it challenging for contrasting our trends with the existing literature. Although our study can demonstrate the potential microbial degradation of the target-pesticide in soil, it is still preliminary on phylogenic profile of bacteria and metabolism are missing out. Thus, further in-depth investigations are necessary to clarify the possible pathways. A possible future broad time-dependent analytical approach would be to reveal the soil-specific microbial world around degradation of tebuthiuron. Another one would be to investigate if it could be possible for N-demethylation^20^ to be a metabolic pathway for native microorganisms to mineralize the target-pesticide into CO_2_. We expect N-demethylation supports the role of tebuthiuron as an electron donor to bacteria as acceptors. Electron donation is an important attribute for the pesticide to be degradable and become harmless or less toxic over an organism, as our ecotoxicological bioassay can describe towards the impact of tebuthiuron on germination and seedling of *L.* *sativa*.

After 90 days respirometry, samples of soil from flasks, where tebuthiuron and bacterial CN-NTS consortium co-exist, could be toxic enough to limit germination or even promote developmental abnormalities, such as stubby radicle or twisting hypocotyl^21^. Hence, seeds can develop into seedlings with radicle-to-hypocotyl ratio closer to 1, which is an indicator of healthy plant^21^. Although ecotoxicological bioassay can cross-validate the potential of rhizospheric native microorganisms to detoxify soil with tebuthiuron, the pesticide could not necessarily be the only source of energy to the microcosm. Flasks containing the target-molecule without isolates, irrespective of composition, can also produce CO_2_. Underlying mechanisms of auto-mineralization of tebuthiuron is unclear, and thus further in-depth investigations are necessary to clarify the fate of the pesticide either directly or through the formation of metabolites to confirm degradation. Interestingly, the sensitive organism can germinate on plates containing soil of 90 days from respirometers, where concentration of tebuthiuron initially range from 2.48 to 4.96 mmol g^−1^ and microbiota does not exist. However, its radicle could be longer/hairier and hypocotyl shorter than normal, which are adaptative response of plant to a stressing microenvironment^22^.

Fitting of kinetic functions is primordial to study mineralization^23^. First-order models are able to predict mineralization. However, they have the limitation of either overfitting or underfitting data on molecules with half-life independent upon time and concentration^23^. If density of degrading microorganisms does not change with time or concentration of pesticide, fitting of first-order functions becomes rather complex and inaccurate. However, as pesticides often sustain growth of degraders, mineralization curves for growing microbial populations are sigmoidal rather than linear^23^. Thereby, we screen Gompertz out of similar microbial growth models^24^ as an option to first-order functions for fitting of mineralization of tebuthiuron into CO_2_. Certainly, sigmoidal Gompertz function can adequately describe the microbial mineralization of tebuthiuron throughout respirometric bioassay, although it cannot fit nongrowth regions and numerous samples could make it challenging for interpreting its parameters. Overall, Gompertz function and its variation^25^ can prove useful to stochastic microbiological studies by predicting degradation^26^, mineralization^27–29^ and ecotoxicology of pollutants/contaminants with accuracy. Therefore, it offers an excellent option for regulatory agencies, researchers and policymakers to replace first-order kinetics in evaluating and elaborating approval procedures for pesticides.

Definitely, the innovative strategy of isolating native rhizospheric microorganisms from areas producing sugarcane with history of exposure to tebuthiuron can prove useful to develop a functional pesticide-degrading framework. Our timely exploratory study introduces the microbial biodegradation of tebuthiuron in agricultural soil. However, it is still preliminary. Thus, further in-depth research is needed to explore the composition and function of the tebuthiuron-degrading bacterial pool and to clarify how the soil changes as the microbiota synergistically and dynamically reduces the target-pollutant into simpler compounds over time. Furthermore, future studies should focus on the microbial metabolism to elucidate the possible genes, enzymes, and pathways controlling the catabolism of tebuthiuron to cross-validate and add information to our preliminary analytical insights into the respirometric-ecotoxicological ramifications of biodegradation. Most importantly, researchers should analyze whether an eventual metabolite could be more toxic and recalcitrant than the parent molecule in order to develop an environmentally safe and responsive microbial bioremediation, which should include an holistical ecotoxicological approach.

## Conclusion

Our preliminary study clearly demonstrates the microbial degradation of tebuthiuron in soil. Native microorganisms from sugarcane’s rhizosphere prove useful to elaborate a synergistic bacterial pool able to produce 89.60 mg CO_2_ day^−1^ upon the target-pesticide at 4.96 mmol g^−1^. The composite inoculum is likely to require 25 days to stabilize the sigmoidal biotransformation on Gompertz function. Our insights provide knowledge of particular relevance to progress in the field’s prominence in microbiologically remediating terrestrial ecosystems, where residual tebuthiuron can persist and contaminate.

## Supplementary Information

Supplementary Table S1.

## Data Availability

The datasets used and/or analyzed during the current study available from the corresponding author on reasonable request. Moreover, they are preserved in the cloud in the Repository Institutional by Unesp, whose periodic backup system promotes greater security.
