# Supplementary material for: A synergistic bacterial pool decomposes tebuthiuron in soil
Source: Sci Rep. 2022 Jun 2;12:9225. doi: 10.1038/s41598-022-13147-8 (PMC9163133; doi:10.1038/s41598-022-13147-8)
Supplement: Supplementary file 1 — Supplementary Table S1. [file 41598_2022_13147_MOESM1_ESM.docx]

Table S1. Physicochemical properties of soil for the respirometric bioassay

| Property | Unit |
| --- | --- |
| pH | 5.20 |
| Organic matter, mg m^-3^ | 3.00 |
| Phosphorus, mmol^c^ dm^-3^ | 5.00 |
| Potassium, mmol^c^ dm^-3^ | 1.70 |
| Calcium, mmol^c^ dm^-3^ | 15.00 |
| Magnesium, mmol^c^ dm^-3^ | 4.00 |
| Potential acidity, mmol^c^ dm^-3^ | 13.00 |
| Exchangeable cations, mmol^c^ dm^-3^ | 21.00 |
| Cation exchange capacity, mmol^c^ dm^-3^ | 34.00 |
| Saturation of exchangeable cations, % | 61.00 |
| Boron, mg dm^-3^ | 0.15 |
| Cupper, mg dm^-3^ | 0.50 |
| Iron, mg dm^-3^ | 8.00 |
| Manganese, mg dm^-3^ | 7.80 |
| Zinc, mg dm^-3^ | 0.30 |
